# The SARS-CoV-2 Vaccine Hesitancy Among the General Population: A Large Cross-Sectional Study From Kuwait

**DOI:** 10.7759/cureus.16261

**Published:** 2021-07-08

**Authors:** Waleed Burhamah, Abdulaziz AlKhayyat, Melinda Oroszlányová, Ahmad AlKenane, Hana Jafar, Mousa Behbehani, Abdulrahman Almansouri

**Affiliations:** 1 Medicine, Royal College of Surgeons in Ireland, Dublin, IRL; 2 Internal Medicine, University of Toronto, Ontario, CAN; 3 Statistics and Data Analysis, College of Engineering and Technology, American University of the Middle East, Kuwait, KWT; 4 General Surgery, Mubarak Alkabeer Hospital, Kuwait, KWT; 5 Psychiatry, University of Toronto, Ontario, CAN; 6 Neurosurgery, McGill University Health Centre, Montreal, CAN

**Keywords:** covid-19, vaccine, pandemic, vaccine hesitancy, vaccine uptake

## Abstract

Although the approved COVID-19 vaccines have proven to be safe and effective, multiple beliefs and misconceptions still exist influencing the vaccine uptake rates around the world. The multifaceted complex phenomenon of vaccine hesitancy could jeopardize the efforts to overcome this pandemic. The aim of this study is to identify the prevalence and examine the factors associated with vaccine hesitancy in Kuwait. This is a web-based cross-sectional study conducted in Kuwait from March 2021 until April 2021, during the second wave of the COVID-19 pandemic. Our questionnaire examined basic demographic information, attitudes towards the COVID-19 vaccines as well as reasons for and against accepting the vaccine. Out of the 2345 responders, the majority are fully convinced to take the vaccine (83%) and the rate of vaccine hesitancy is 17%. Vaccine hesitancy is higher among non-healthcare workers, those previously positive for the COVID-19 virus, and those against vaccines in general. Vaccine hesitancy could jeopardize the efforts to overcome this pandemic; therefore, intensifying nationwide education and dismissal of falsified information is an essential step towards addressing vaccine hesitancy.

## Introduction

Despite the multiple efforts and strategies to reduce the spread of the severe acute respiratory syndrome coronavirus 2 (SARS-CoV-2), it remains today a global health pandemic since December 2019, responsible for more than 131 million cases and more than 3 million deaths worldwide [1]. Among the detrimental physical, economic, and psychosocial sequelae that have arisen as a consequence [2-4], it was clear that vaccination was a necessary step towards ending this pandemic. Many countries embarked on a mission to develop vaccines at unsurpassed efforts. On December 11, 2020, the United States Food and Drug Administration (FDA) issued an emergency use authorization for the Pfizer-BioNTech COVID-19 vaccine [5], and only a week later, the Moderna COVID-19 vaccine received emergency use authorization [6]. The third approval came on February 27, 2021, when the FDA issued an emergency approval and use authorization for the Janssen COVID-19 vaccine. Unlike the previous two vaccines, the Janssen vaccine consists of only a single dose [7]. The Oxford-AstraZeneca vaccine was authorized on January 29, 2021, receiving approval from the European Medicines Agency [8]. In December 2020, Kuwait has begun its vaccination campaign where two vaccines were made available, the Pfizer-BioNTech [9] and the Oxford-AstraZeneca [10,11].

Vaccine hesitancy is a long-known concept to mankind, listed as one of the top 10 threats to global health by the World Health Organization (WHO) [12]. It is of no surprise that such a notion will re-emerge to halt the progress of achieving herd immunity. It is estimated that at least 67% of the population is required to be vaccinated in order to achieve herd immunity [13].

Unfortunately, there seems to be a worldwide skepticism surrounding the COVID-19 vaccines resulting in hesitancy and low uptake rates [14,15]. Multiple beliefs and misconceptions are behind the hesitancy, for example, alleged uncertainty regarding the long-term adverse events of the vaccines as well as multiple conspiracy theories [14,15]. Conspiracy theories surrounding this pandemic started early on, one being around the virus being man-made, created as a gateway to developing vaccines consisting of microchips intended to control humans [14].

With the continued circulation of such misleading theories, negative perception of the COVID-19 vaccines in the general public seemed quite inevitable. A systematic review by Sallam M et al showed a low vaccine acceptance rate in the Middle East [16]. Similarly, the rates of vaccine hesitancy in Italy were shown to be as high as 40.1% of the studied population [17]. These alarming rates of vaccine hesitancy are likely to pose a major threat to the attempts of overcoming this pandemic.

The aim of the study is to identify the prevalence of vaccine acceptance and hesitancy among our studied cohort and to determine the factors associated with vaccine hesitancy.

## Materials and methods

Study design and participants

This is a cross-sectional study conducted in Kuwait from the period of March 2021 until April 2021. This period corresponds to the second wave of the COVID-19 pandemic whereby lockdown measures had been re-introduced. A web-based questionnaire was sent on various social media platforms targeting adults above the age of 18 years who were living in Kuwait. The link to the online questionnaire was supplemented with a message detailing the aim of the questionnaire, that it is confidential and anonymous, and that participation is optional. Informed consent was obtained from the responders prior to progressing to the questionnaire. The questionnaire was distributed by one of the authors who is not a healthcare professional. This was done in an attempt to minimize response bias that could potentially arise if a medical doctor were to distribute the questionnaire. Ethical approval was obtained from the ethical committee in the Ministry of Health in Kuwait prior to the distribution of the questionnaire.

Variables

The questionnaire examined basic demographic information as well as attitudes towards the COVID-19 vaccines. Basic demographic information was obtained including age, weight, height, gender, smoking status, exercise frequency, educational background, and presence of medical co-morbidities. Participants were asked if they were healthcare workers (this includes those in the medical field and working in a hospital) - doctors of all specialties, physiotherapists, pharmacists, dentists, and nutritionists. The participants were also asked if they were against vaccines in general, if they were previously positive for the COVID-19 virus, and if they had a family member and/or a close friend who passed away from the COVID-19 virus.

To investigate the attitudes towards the COVID-19 vaccines, the participants were asked the question - ‘Which of the following statements describes how you feel about the COVID-19 vaccines?’ In those who are accepting to take the vaccine, the following question was given: ‘Which of the following reason(s) describes why you are choosing to take the COVID-19 vaccine? (You may pick more than one answer)’. In those responders who are either against or skeptical about the vaccine, the following question was provided: ‘Which of the following reason(s) explains why you are hesitant about taking the COVID-19 vaccine? (You may pick more than one answer)’. To examine the participants’ knowledge about the vaccine, they were asked when do they think the vaccine will start taking effect: ‘After taking the COVID-19 vaccine, how soon do you think it will start working?’

Statistical analysis

The statistical analysis was performed using R software, version 4.0.5 (R Foundation, Vienna, Austria). The continuous variables were reported as median (interquartile range - IQR), and categorical variables were reported as a frequency (percentage). To determine the factors associated with vaccine hesitancy, the continuous variables were tested by the t-test and Mann-Whitney U test (at α = 0.05), and the categorical variables were tested by Chi-squared test (at α = 0.05). The odds ratios (OR) were calculated with the 95% confidence intervals (CI) and the corresponding p-values.

## Results

Demographics

A total of 2345 individuals participated in this study. The socio-demographic results are summarised in Table 1. The median age of participants was 29, with more than half of the individuals being unmarried (55%), smokers (78%), of female gender (59%), and with a bachelor’s degree (55%). A relatively small proportion was against vaccinations in general (5.1%). Only (18%) were previously infected with the COVID-19 virus, and 0.1% were admitted to the intensive care unit (ICU). Some of the participants had a family member and/or a close friend who passed away from the COVID-19 virus (38%). Most of the participants received one dose of the COVID-19 vaccine (46%), 26% were fully vaccinated with two doses, 9% registered and were waiting to be vaccinated while 19% did not register to receive the vaccine (Table 2).

**Table 1:** Demographics of the survey participants. *Median (interquartile range); n (%)

Characteristic	N = 2,345*
Age	29 (23, 40)
Weight	72 (60, 86)
Height	165 (159, 173)
BMI	26.0 (22.7, 30.1)
Married	
Yes	1,050 (45%)
No	1,295 (55%)
Do you smoke?	
Yes	514 (22%)
No	1,831 (78%)
Gender	
Female	1,393 (59%)
Male	952 (41%)
Exercise frequency	
1-3 times per week	760 (32%)
3-4 times per week	319 (14%)
>5 times per week	263 (11%)
0 times per week	1,003 (43%)
Education obtained	
High school	481 (20%)
College diploma	323 (14%)
Bachelor’s degree	1,290 (55%)
Master’s degree/PhD	251 (11%)
Are you a healthcare worker?	
Yes	329 (14%)
No	2,016 (86%)
Diabetes	
Yes	168 (7.2%)
No	2,177 (92.8%)
Hypertension	
Yes	242 (10%)
No	2,103 (90%)
Have you had a previous heart attack or stroke?	
Yes	27 (1.2%)
No	2,318 (98.8%)
Asthma	
Yes	168 (7.2%)
No	2,177 (92.8%)
Hypothyroidism	
Yes	55 (2.3%)
No	2,290 (97.7%)
Anxiety/Depression	
Yes	16 (0.7%)
No	2,329 (99.3%)
Cancer	
Yes	6 (0.26%)
No	2,339 (99.74%)
Rheumatologic condition (RA SLE Psoriasis)	
Yes	24 (1%)
No	2,321 (99%)
Inflammatory bowel disease (IBD)	
Yes	14 (0.6%)
No	2,331 (99.4%)
Number of medical conditions	
0	1,779 (76%)
1	432 (18%)
2	115 (4.9%)
3	18 (0.8%)
4	1 (<0.1%)
Were you previously positive for the COVID-19 virus?	
Yes	412 (18%)
No	1933 (82%)
Are you against vaccines in general?	
Yes	120 (5.1%)
No	2,225 (94.9%)
After taking the COVID-19 vaccine, how soon do you think it will start working?	
As soon as I take the vaccine	148 (6.3%)
After 1-6 days	227 (9.7%)
After 1 week	263 (11%)
Between 2-3 weeks	1,306 (56%)
More than 4 weeks	401 (17%)
Have you had a family member/friend who passed away from the COVID-19 Virus?	
Yes	880 (38%)
No	1,465 (62%)

**Table 2:** The prevalence of vaccinated individuals. *N (%)

‘Have you received the COVID-19 vaccine?’	N = 2,345^*^
Yes, 1 dose only	1,075 (46%)
Yes, 2 doses	600 (26%)
Not yet, but I registered	221 (9%)
No, and I have not registered	449 (19%)

Attitudes towards the COVID-19 vaccine

Table 3 summarises the responders’ attitudes towards the COVID-19 vaccines. The majority of the participants were fully convinced they should take the vaccine (1955/2345, 83%). On the other end of the spectrum (299/2345, 12.8%) were those against the COVID-19 vaccine and will not take it. Only 4.2%(91/2345) were not against the vaccine but will not take it because they are worried. Therefore, the rate of vaccine hesitancy [17] in our cohort is 17% (N=390 [299+91]). The most common reasons for not taking the COVID-19 vaccine are summarized in Table 4. The most common reason among those who were accepting of the vaccine (and had taken or were willing to take the vaccine (1955/2345, 83%) was that so the pandemic ends faster and life returns to normal (70.3%). The second most common reason was protection against the virus (53.2%) and 33.4% wanted to be able to travel in the future (Table 5).

**Table 3:** Attitudes towards the COVID-19 vaccines. *N (%)

‘Which of the following statements describes how you feel about the COVID-19 vaccines?’	N = 2,345^*^
I am against the COVID-19 vaccine and will not take it.	299 (12.8%)
I am not against the COVID-19 vaccine and I am fully convinced I should take it.	1,955 (83%)
I am not against the COVID-19 vaccine but I will not take it because I am worried or hesitant.	91 (4.2%)

**Table 4:** The most common reasons for not taking the COVID-19 vaccine.

‘Which of the following reason(s) explains why you are hesitant about taking the COVID-19 Vaccine? (You may pick more than one answer)’	N = 390^1^
I am waiting to see how it affects other people	385 (98.7%)
Because it is not fully protective against the virus	130 (33.3%)
I think it is a conspiracy	106 (27.2%)
I think it is harmful and/or unsafe	72 (18.5%)
I think the vaccine can give me the COVID-19 virus	36 (9.2%)

**Table 5:** The most common reasons for taking the COVID-19 vaccine. *N (%)

‘Which of the following reason(s) describes why you are choosing to take the COVID-19 Vaccine?’ (You may pick more than one answer).	N = 2,345^*^
So that the pandemic ends faster and life returns to normal.	1,629 (70.32%)
To protect me against the virus.	1,229 (53.22%)
So that I can travel in the future.	765 (33.43%)
I will not/might not take the vaccine.	390 (14.16%)

Patient education regarding the vaccine

More than half of the participants (56%) were correct in identifying when the vaccine usually reaches its optimal level of protection (two to three weeks from receiving the vaccine), 17% thought it will start working only after more than four weeks, and 11% thought it will work after one week. A few of the participants thought that the vaccine will start working after one to six days (9.7%) or as soon as it is taken (6.3%).

Factors associated with being against the COVID-19 vaccine: As summarized in Table 6, a number of statistically significant associations were observed. 

**Table 6:** Factors associated with being against the COVID-19 vaccines. OR: odds ratio; Ref.: reference

	Against vaccine (N=299)	Not against vaccine (N=2046)	OR	p-value (OR)	p-value (overall)
Age	34.0 (12.7)	32.2 (12.6)	0.99 [0.98;1.00]	0.023	0.024
BMI	26.9 (6.64)	27.3 (8.75)	1.01 [0.99;1.03]	0.419	0.333
Married					0.007
Yes	156 (52.2%)	894 (43.7%)	Ref.	Ref.	
No	143 (47.8%)	1152 (56.3%)	1.41 [1.10;1.79]	0.006	
Do you smoke?					0.233
Yes	74 (24.7%)	440 (21.5%)	Ref.	Ref.	
No	225 (75.3%)	1606 (78.5%)	1.20 [0.90;1.59]	0.209	
Gender					0.17
Female	189 (63.2%)	1204 (58.8%)	Ref.	Ref.	
Male	110 (36.8%)	842 (41.2%)	1.20 [0.94;1.55]	0.151	
Education obtained					0.285
Highchool	58 (19.4%)	423 (20.7%)	Ref.	Ref.	
College Diploma	52 (17.4%)	271 (13.2%)	0.71 [0.48;1.07]	0.105	
Bachelor’s Degree	158 (52.8%)	1132 (55.3%)	0.98 [0.71;1.35]	0.921	
Master’s/PhD	31 (10.4%)	220 (10.8%)	0.97 [0.61;1.56]	0.902	
Are you a healthcare worker?					0.026
Yes	29 (9.70%)	300 (14.7%)	Ref.	Ref.	
No	270 (90.3%)	1746 (85.3%)	0.63 [0.41;0.92]	0.017	
Diabetes					0.089
Yes	29 (9.70%)	139 (6.79%)	Ref.	Ref.	
No	270 (90.3%)	1907 (93.2%)	1.48 [0.95;2.22]	0.079	
Hypertension					0.783
Yes	29 (9.70%)	213 (10.4%)	Ref.	Ref.	
No	270 (90.3%)	1833 (89.6%)	0.93 [0.61;1.38]	0.72	
Previous heart attack or stroke					0.144
Yes	6 (2.01%)	21 (1.03%)	Ref.	Ref.	
No	293 (98.0%)	2025 (99.0%)	2.01 [0.72;4.77]	0.167	
Asthma					0.032
Yes	12 (4.01%)	156 (7.62%)	Ref.	Ref.	
No	287 (96.0%)	1890 (92.4%)	0.51 [0.27;0.90]	0.018	
Hypothyroidism					0.536
Yes	5 (1.67%)	50 (2.44%)	Ref.	Ref.	
No	294 (98.3%)	1996 (97.6%)	0.70 [0.24;1.61]	0.429	
Anxiety/Depression					0.137
Yes	4 (1.34%)	12 (0.59%)	Ref.	Ref.	
No	295 (98.7%)	2034 (99.4%)	2.36 [0.64;6.91]	0.18	
Cancer					0.559
Yes	1 (0.33%)	5 (0.24%)	Ref.	Ref.	
No	298 (99.7%)	2041 (99.8%)	1.52 [0.06;9.93]	0.731	
Rheumatological (RA/SLE/Psoriasis)					0.112
Yes	6 (2.01%)	18 (0.88%)	Ref.	Ref.	
No	293 (98.0%)	2028 (99.1%)	2.35 [0.83;5.69]	0.101	
Inflammatory bowel disease (IBD)					0.092
Yes	4 (1.34%)	10 (0.49%)	Ref.	Ref.	
No	295 (98.7%)	2036 (99.5%)	2.82 [0.75;8.65]	0.116	
Were you previously positive for the COVID-19 virus?					0.006
Yes	70 (23.4%)	342 (16.7%)	Ref.	Ref.	
No	229 (76.6%)	1704 (83.3%)	1.52 [1.13;2.03]	0.006	
Were you admitted to the intensive care unit? (ICU)					0.045
Yes	2 (0.67%)	1 (0.05%)	Ref.	Ref.	
No	297 (99.3%)	2045 (100.0%)	12.9 [1.04;406]	0.047	
Are you against vaccines in general?					<0.001
Yes	94 (31.4%)	26 (1.27%)	Ref.	Ref.	
No	205 (68.6%)	2020 (98.7%)	35.4 [22.7;56.9]	0	
After taking the COVID-19 vaccine, how soon do you think it will start working?					<0.001
As soon as you take the vaccine	35 (11.7%)	113 (5.52%)	Ref.	Ref.	
After 1-6 days	31 (10.4%)	196 (9.58%)	1.95 [1.14;3.36]	0.015	
After 1 week	36 (12.0%)	227 (11.1%)	1.95 [1.16;3.28]	0.012	
Between 2-3 weeks	110 (36.8%)	1196 (58.5%)	3.37 [2.18;5.13]	<0.001	
More than 4 weeks	87 (29.1%)	314 (15.3%)	1.12 [0.71;1.74]	0.622	
Have you had a family member/friend who passed away from the COVID-19 Virus?					0.636
Yes	108 (36.1%)	772 (37.7%)	Ref.	Ref.	
No	191 (63.9%)	1274 (62.3%)	0.93 [0.72;1.20]	0.594	
Co-mobidities					1
Yes	227 (75.9%)	1552 (75.9%)	Ref.	Ref.	
No	72 (24.1%)	494 (24.1%)	1.00 [0.76;1.34]	0.988	

Age (OR = 0.99): Although the test results show a significant difference in the average age between those against the COVID-19 vaccine and those not against the vaccine (overall p-value < 0.05), the odds ratio is almost equal to 1.

Marital status (OR = 1.41): Individuals against the COVID-19 vaccine are 41% more likely to be married than those who are not against the vaccine.

Healthcare workers (OR = 0.63): Individuals against the COVID-19 vaccine are 37% less likely to be healthcare workers than those not against the vaccine. Hence being a healthcare worker is associated with higher vaccine acceptance.

Asthma (OR = 0.51): Individuals against the COVID-19 vaccine are 49% less likely to have asthma than those not against the vaccine. Asthma is associated with higher vaccine acceptance.

Being previously positive for the COVID-19 virus (OR = 1.52): Those against the COVID-19 vaccine are 52% more likely to have been previously positive for the COVID-19 virus than those who aren’t against the vaccine.

Being admitted to the intensive care unit (ICU) (OR = 12.9): Individuals against the COVID-19 vaccine are 12.9 times more likely to have been admitted to the ICU than those not against the vaccine.

Being against vaccines in general (OR = 35.4): Individuals against the COVID-19 vaccine are 35.4 times more likely to be against vaccines in general than those who aren’t against the COVID-19 vaccine.

Expected time for the vaccine to start working (OR=3.37): Individuals against the COVID-19 vaccine are 3.37 times more likely to think that the vaccine will start working as soon as it is taken (in comparison to after two to three weeks) than those not against the COVID-19 vaccine.

## Discussion

Vaccine hesitancy is defined as the “delay in acceptance or refusal of safe vaccines despite availability of vaccination services” [18]. The phenomenon of vaccine hesitancy is complex, with multiple factors coming into play - cognitive, psychological, socio-demographic, and cultural. Generally, the determinants of vaccine acceptance fall under three main categories - complacency, convenience, and confidence [18]. Complacency is related to the perception of the risk of acquiring the disease for which the vaccine is being given. Confidence refers to trust in the healthcare system, vaccination safety, and efficacy. Convenience is the delivery of vaccines in an affordable, accessible, and comfortable fashion [16]. Understanding such concepts can provide areas of improvement to combat vaccine hesitancy and ultimately increase acceptance rates.

The rates of hesitancy towards the COVID-19 vaccine have been shown to vary widely across countries. Many predictors of increased vaccine hesitancy have been identified, including but not limited to demographic characteristics, level of education, ethnicity, the perceived likelihood of acquiring the COVID-19 virus, and affordability of the vaccine [19-21]. Currently, the ultimate goal is to overcome this pandemic, and with the availability of many approved vaccines, this can be achieved with herd immunity. This concept is based on the theory of vaccinating an adequate proportion of a given population to an extent that is enough to limit the spread of a virus, inevitably offering protection to those who remain unimmunized. It is estimated that for the COVID-19 virus, vaccine uptake would need to be between 67% and 80% to achieve herd immunity [22]. Unfortunately, with those hesitant towards the vaccine, the efforts to end this pandemic could be in jeopardy. 

Large heterogeneity in vaccine acceptance rates is seen in the literature. In a literature review by Sallam et al., acceptance rates of more than 90% were seen in Indonesia, Malaysia, and China [23,24], while in Singapore and India, the rates were relatively lower (67.9% and 74.5%, respectively) [25]. Acceptance rates from countries in Europe also varied widely, with rates ranging from 53.7% to 58.9% in Italy, France, Poland, and Russia [16,26] and higher rates of up to 80.0% reported from Denmark [27]. However, in Latin America, vaccine acceptance rates were more consistent, showing rates of more than 70% in Brazil, Ecuador, and Mexico [16,28] Unfortunately data from the continent of Africa is limited, with two studies reporting an acceptance rate of 81.6% in South Africa and 65.2% in Nigeria [16,25].

In our cohort, the rate of vaccine hesitancy (those who refuse to take the vaccine and those who are skeptical) [18] was found to be 17% (Table 4). The most common reason as to why participants were hesitant towards the vaccine was ‘waiting to see how it affected other people’. Similarly in a recent review examining 15 studies surrounding vaccine hesitancy, many had concerns about the safety of the vaccines, perceived them as rushed, and with an unknown safety profile [29]. The second most common reason in our participants was ‘thinking that the vaccine is not fully protective against the virus’. The aforementioned reasons indicate the lack of understanding and awareness in this subgroup regarding the regulated and rigorous process involved in developing a vaccine, to ensure safety and efficacy prior to approval and distribution. Such lack of knowledge can be addressed by raising awareness about vaccines via social media platforms using clear and simple language. The third most common reason for being hesitant towards the vaccine was ‘thinking that it is a conspiracy’. Unfortunately, such misconception has been circulating on multiple social media platforms and has been shown to hinder the uptake of vaccines [14,15]. This can be addressed by creating short videos and documentaries referring to the history of pandemics faced by mankind since pandemics have occurred in the past and their occurrence is unintentional. 

From our responders, we found an acceptance rate of 83%. Looking at reasons behind the willingness to accept the COVID-19 vaccines (Table 3), the most common reason was ‘so that the pandemic ends faster and life returns back to normal’ while ‘protection against the virus’ was only the second most common reason. This shows that a high proportion of those receiving the vaccine was not too concerned with the implications of being infected but wanted life to return back to normal as a priority. Interestingly, a previous study based on a cohort from Kuwait showed a much lower acceptance rate [15]; however, our current study was undertaken during the second wave after the reintroduction of the lockdown measures. This could correlate with the higher rate of vaccine acceptance during the lockdown, which was also seen in a recent study in Italy [17].

Healthcare workers exhibited a significantly lower rate of vaccine hesitancy. A higher perceived risk of acquiring the disease, knowledge regarding the severity of the disease, and the physiology of vaccination could be contributory factors. Similar results of higher vaccine uptake rates amongst healthcare workers were found in several other studies [30,31]. Chew et al concluded that a higher perception of viral susceptibility, lower vaccine harm index, and high pro-socialness index were independent predictors for better vaccine acceptance rates among healthcare workers [32]. We found that participants who were against vaccines in general were significantly more likely to be against the COVID-19 vaccine in specific. Anti-vaccine campaigns have existed prior to this pandemic, and the rise of social media not only made it easier for such beliefs to gain popularity but also falsified information regarding the safety of the vaccine has multiplied and spread at a faster rate than ever [14].

No doubt the Internet has played a large role in vaccine hesitancy - and contrary to what some believe, the burden of the pandemic has not been enough to dissuade anti-vaccine searches. In fact, anti-vaccine searches questioning the safety of vaccines have continued to grow during this pandemic [33]. This shows the power that the Internet holds in influencing a wide range of audiences, and hence, should be utilized as a tool to raise awareness, discredit falsified information, and misconceptions.

Our study also showed that participants who previously acquired the COVID-19 virus were more likely to be against vaccination. A possible explanation could be the false belief of acquiring indefinite immunity to the virus given prior infection and lack of knowledge regarding the possibility of re-infection.

Interestingly, having a chronic medical condition did not seem to reduce vaccine hesitancy - this is contrary to what is expected. It is a well-established observation in those affected by the COVID-19 virus that the presence of co-morbidities confers an unfavorable outcome. As a result, we expected higher acceptance rates among those with co-morbidities. This unexpected finding was also seen in a similar study published in the United States [34].

We examined the participants’ knowledge about the COVID-19 vaccine by asking them: ‘After taking the COVID-19 vaccine, how soon do you think it will start working?’ This question was answered on multiple occasions by members of the vaccination committee in Kuwait as well as being available in multiple leaflets. Interestingly, individuals against the COVID-19 vaccine were 3.37 times more likely to answer incorrectly than those not against the vaccine. This shows that a general lack of knowledge regarding the vaccine and its efficacy is highly associated with refusal to accept the vaccine.

Limitations

A number of limitations exist in our study. Due to the nature of this cross-sectional study and the delivery of the questionnaire by an online mode, our study is subject to selection bias. We attempted to minimize this by spreading our questionnaire over multiple social media platforms, text messages, and online feeds. Although participants of multiple backgrounds were captured, being an online survey inevitably excluded those of an older age group who are not users of such online platforms. Lastly, although our study examined some potential socio-demographic determinants of vaccine acceptance rates and hesitancy, other potential factors have not been examined. These include - the perceived risk of acquiring the virus, the perceived severity of the disease, the source of acquiring information regarding the COVID-19 vaccine, affordability, ethnicity, and level of trust in the local healthcare system.

## Conclusions

As a result of the COVID-19 pandemic, the lives of many have been adversely affected on multiple dimensions - physically, psychologically, and/or economically. Vaccine hesitancy is no doubt an obstacle to overcoming this pandemic. We found high rates of vaccine hesitancy among non-healthcare workers, those previously positive for the COVID-19 virus, and those against vaccines in general. Multiple conspiracy theories have existed since the beginning of this pandemic. The current literature provides evidence that rates of vaccine hesitancy are increased amongst those with higher exposure to social media and supporters of the conspiracy theories. Up to one-third of our cohort are supporters of such conspiracy theories. Although the influence of social media has not been investigated in our cohort, channeling social media platforms into raising awareness and dismissal of falsified information could be a potential means of addressing vaccine hesitancy.
